# Supplementary material for: Novel Monoclonal Antibodies 1D2 and 4E4 Against Aspergillus Glycoprotein Antigens Detect Early Invasive Aspergillosis in Mice
Source: J Fungi (Basel). 2024 Dec 2;10(12):832. doi: 10.3390/jof10120832 (PMC11678807; doi:10.3390/jof10120832)
Supplement: Supplementary file 1 [file jof-10-00832-s001.zip › jof-3351048-supplementary.pdf]

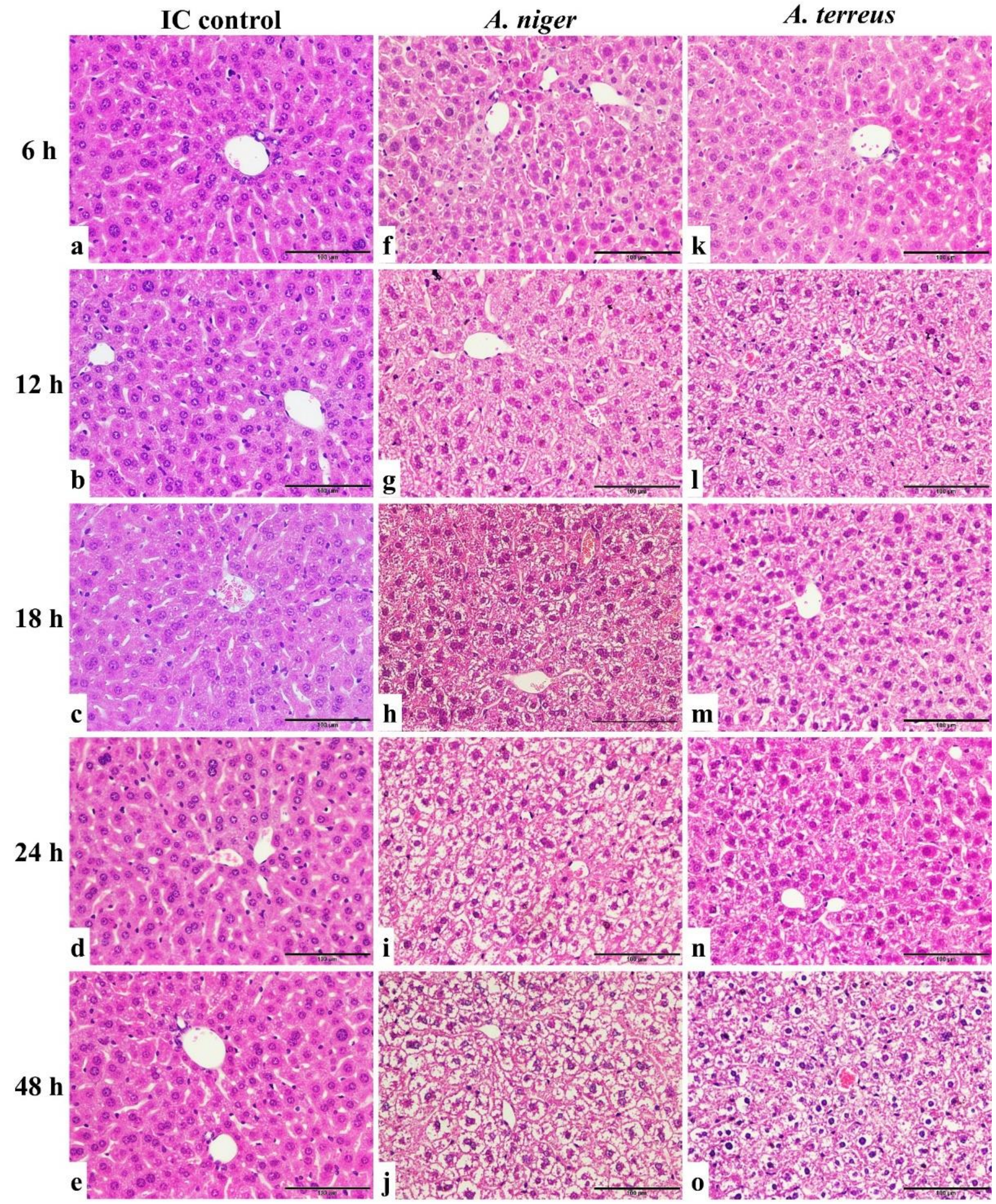

Figure S1 HE staining of liver sections of *A. niger* and *A. terreus* infected mice.

The liver of immunocompromised (IC) control mouse did not show significant injuries in tissue (a-e). For *A. niger* (f-j) and *A. terreus* (k-o) infected mice, there was no significant necrosis in the tissues six hours post infection (panel f and k). From 12 to 24 hours (g-i and l-n), the liver progressively showed cytoplasmic vacuolation and necrosis of hepatocytes. Forty-eight hours post infection (j and 0), the tissues showed progressive cytoplasmic vacuolation and necrosis but no hyphal infiltration in liver. Scale bars represent 100 μm.
